# Ophthalmic Manifestations in Patients with Blood Malignancies

**DOI:** 10.3390/hematolrep16020020

**Published:** 2024-03-28

**Authors:** Costanza Rossi, Alessandro Buizza, Giuseppe Alessio, Massimiliano Borselli, Andrea Taloni, Adriano Carnevali, Giovanna Carnovale Scalzo, Andrea Lucisano, Vincenzo Scorcia, Giuseppe Giannaccare

**Affiliations:** 1 Department of Ophthalmology, University Magna Graecia of Catanzaro, 88100 Catanzaro, Italy; costanzarossi9@gmail.com (C.R.); giuseppe.alessio001@studenti.unicz.it (G.A.); mborselli93@gmail.com (M.B.); taloni.oculistica@gmail.com (A.T.); adrianocarnevali@unicz.it (A.C.); giovannacarnovalescalzo@unicz.it (G.C.S.); andrealucisano@alice.it (A.L.); vscorcia@unicz.it (V.S.); 2 Department of Biomedical Sciences, Humanitas University, via Rita Levi Montalcini 4, 20072 Milan, Italy; alessandro.buizza@humanitas.it; 3 IRCCS Humanitas Research Hospital, via Manzoni 56, 20089 Milan, Italy; 4 Eye Clinic, Department of Surgical Sciences, University of Cagliari, 09123 Cagliari, Italy

**Keywords:** hematological malignancies, ocular complications, leukemic retinopathy, ocular graft versus host disease, antineoplastic therapy toxicity

## Abstract

Ocular complications can occur in up to 90% of patients with blood malignancies. Such complications range from direct infiltration to local hemostatic imbalance and treatment-related toxicity. This narrative review is based on a systematic computerized search of the literature conducted until January 2024 and examines the common ocular complications associated with blood cancers. Ocular complications from primary disease include mass effects from ocular adnexal lymphomas and intraocular lymphomas, with B-cell lymphomas accounting for 95% of primary ocular presentations. Secondary disease involvement from systemic hematological malignancies can lead to a wide range of ocular manifestations, such as leukemic retinopathy. Furthermore, toxicity from antineoplastic therapies and ocular graft versus host disease (oGVHD) after hematopoietic stem cell transplantation present additional risks to ocular health. In conclusion, ocular complications in blood cancer patients are an integral part of patient management, requiring regular ophthalmic evaluations and close collaboration between oncologists and ophthalmologists. Advances in therapy and an increased focus on early symptom recognition are essential for preserving vision and enhancing patient quality of life.

## 1. Introduction

Hematological malignancies represent a set of blood disorders affecting the lymphoid or myeloid lineage. The most frequently reported manifestations of the disease include weight loss, fatigue, and fever; however, eye changes may be observed in up to 90% of affected individuals, depending on the underlying condition [1,2]. All ocular structures may be affected due to a variety of pathological mechanisms, including direct infiltration, local hemostatic imbalance, and treatment-related direct toxicity [3]. Ocular graft versus host disease (GVHD) after hematopoietic stem cell transplantation (HSCT) represents another important complication of cancer therapy, requiring prompt ophthalmological evaluation and treatment [3,4]. Finally, lymphoproliferative diseases may affect the eye primarily as extranodal lymphomas of the orbit, ocular adnexa, and vitreoretinal tissue [5,6,7].

Despite a growing number of reported cases, eye manifestations of blood cancers are relatively unknown for the clinician and may masquerade as common disorders. This narrative review outlines the most common ocular complications observed in patients with hematological malignancies.

## 2. Primary Disease Involvement of the Eye

Ophthalmologic complications related to hematologic diseases often arise due to primary or secondary involvement of the eye or the ocular adnexa. Reasonably, the primary manifestation of such involvement is directly correlated with the presence of the disease, and the symptoms vary depending on the anatomical structure affected.

Primary or secondary involvement of the eye is considered rare in leukemias as well as an uncommon occurrence in lymphomas. Regardless of the rarity of such lymphomas, a stable increase in incidence during the last decades has been observed, with an annual change of 4.8% from 1973 to 2009 [8]. Approximately 95% of blood tumors primarily affecting the eye are B-cell lymphomas, with only a few cases of T-cell lymphomas, of which the majority are NK T-cell lymphomas [9]. Most cases of ocular lymphomas are of primary involvement of the eye structures, and in only 10–30% of cases, there is a secondary involvement from diffuse lymphomas [10].

Based on their anatomical localization, ocular lymphomas can be classified into ocular adnexal lymphomas and intraocular lymphomas. The first category includes extranodal marginal zone lymphomas (EMZL) (37–68%), follicular lymphomas (FL) (10–23%), diffuse large B-cell lymphomas (DLBCL) (10–15%), and mantle cell lymphomas (MCL) (7–8%) [5]. Rarely also other types, such as Hodgkin lymphoma, Burkitt lymphoma, chronic lymphocytic leukemia, or plasma cell disorders, may involve the eye [5]. Intraocular lymphomas include vitreoretinal lymphomas and secondary intraocular lymphomas.

### 2.1. Ocular Adnexal Lymphomas

Ocular adnexal lymphomas primarily present symptoms associated with their mass effect. These include proptosis, reduced eye motility, pain, ptosis, changes in visual acuity, diplopia, and a palpable mass. Rarely, symptoms are related to the local invasion of adjacent anatomical structures [5,10]. Symptom duration and rapidity of onset are related to the disease nature. Diagnosis is always performed on histological specimens, preferably from excisional biopsy [11]. 

The selection of a treatment plan is primarily guided by factors including the type of lymphoma and its molecular features, the presence of symptoms related to eye involvement, and the extent of disease spread. Typically, in cases of localized disease, radiotherapy is frequently employed, utilizing radiation doses between 20 and 40 Gy to achieve optimal disease control [12,13].

Regarding higher-stage diseases, systemic treatments are preferred owing to the increased risk of disease recurrence. Options include chemotherapy, immunotherapy, or combinations of both, with variable results according to the disease characteristics [14,15,16].

### 2.2. Intraocular Lymphomas

Primary intraocular lymphomas are considered a subset of primary central nervous system lymphomas (PCNS) and have been reported in the literature as vitreoretinal lymphoma or PCNS lymphoma ocular variant [7]. While relatively uncommon, intraocular lymphomas pose unique challenges in terms of diagnosis and management due to their intricate connection with the eye and central nervous systems [17]. 

Primary vitreoretinal lymphoma (PVRL) is a rare form of DLBCL that initially exclusively involves the posterior structures of the eye, i.e., the vitreous body, retina, and rarely the optic nerve, without concomitant central nervous system (CNS) involvement at diagnosis [18,19]. PVRL is often referred to as masquerade syndrome, as its symptoms closely mimic those of posterior uveitis, and it initially responds to steroid treatment [20]. This condition typically presents with bilateral eye involvement in approximately 70% of cases. Common initial symptoms include blurred vision, painless loss of vision, and floaters. Upon examination, a variable degree of vitritis is observed, characterized by the presence of lymphoma cells in the anterior vitreous [21]. In detail, the distribution of malignant cells along the vitreal fibrils gives rise to a characteristic “aurora borealis” appearance [22]. At the level of the retina, lymphoma infiltrates may be evident by multifocal cream-colored retinal spots, mimicking drusen [23]. Anterior chamber findings are uncommon in PVRL; however, cases of occurrences like keratic precipitates, iris or angle infiltration, and, very rarely, pseudohypopyon can also be observed [24,25]. Optic nerve infiltration in systemic metastatic retinal lymphoma may have initial occult signs but with profound visual loss [26]. 

Diagnostic procedures include magnetic resonance imaging (MRI), optical coherence tomography (OCT), fluorescein angiography, positron emission tomography (PET) scan, and assessment of interleukin (IL)-10 levels in the vitreous, but ultimately the diagnosis is made on the histological specimen by vitrectomy [27]. Treatment is heterogeneous, with patients being treated either locally with intravitreal injections of chemotherapies, immunotherapies, or ocular irradiation [17]. 

As PVRL involves the posterior structures of the eye, very few cases of aggressive and indolent lymphomas affect the uvea, most commonly EMZLs involving the choroid, while even rarer cases have been reported of DLBCL affecting the iris [28,29]. The clinical presentation of choroidal lymphoma is characterized by blurred vision or metamorphopsia, as well as salmon-colored subconjunctival patches when transscleral infiltration occurs [30]. Ciliary lymphomas, on the other hand, have a clinical presentation resembling that of anterior uveitis [31]. Notwithstanding the type of lymphoma, involvement of the iris and angle may lead to secondary glaucoma [31]. Prognosis and treatment depend on the histologic type, which has a very good prognosis for EMZL [28]. 

## 3. Secondary Disease Involvement 

Hematological malignancies that may affect the eye include leukemias and lymphomas, as well as multiple myeloma, myeloproliferative neoplasms, myelodysplastic syndromes, and Waldenstrom’s macroglobulinaemia, potentially affecting all eye structures [1,32,33,34,35,36].

Retinal tissue is commonly involved in leukemias, occurring in up to 30–50% of affected individuals [37,38,39]. Leukemic retinopathy is more frequently observed in acute leukemias and is the most common ophthalmic manifestation in affected patients [40,41]. Primary leukemic retinopathy involves direct infiltration of the retina by cancerous leukocytes, while secondary retinopathy results from hematological complications of leukemia, such as thrombocytopenia, anemia, and hyperviscosity [3,42]. The hallmark of leukemic retinopathy is retinal hemorrhages, stemming from both direct infiltration by leukemic cells and the broader impacts of secondary leukemic retinopathy. Hemorrhages may vary in appearance, presenting as dots, Roth spots (white-centered hemorrhages), or flame-shaped patterns, and may extend into the subretinal tissue or vitreous. 

Cotton wool spots are also frequent and result from nerve fiber layer infarcts or localized accumulations of leukemic cells [43]. Moreover, peripheral microaneurysms and neovascularization are significant ocular signs of chronic leukemia, with up to 50% of affected individuals showing these manifestations in their peripheral retina [44]. 

Nodular retinal infiltrates occur in association with elevated leukocyte counts and are the result of leukostasis and direct retinal infiltration in both acute and chronic pathologies [45,46]. Infiltrates are often described as grayish-white and may involve the foveal area, affecting vision [47,48]. 

Bilateral or mono-lateral serous detachment of the retina is associated with diffuse infiltration of leukemic cells within and surrounding choroidal vessels [49,50]. Possibly, blood stagnation or mechanical compression of choroidal vessels results in ischemia of the overlying retinal pigment epithelium and disruption of the intercellular tight junctions [50,51]. 

Central retinal vein and/or artery occlusion is a rare manifestation of leukemia and often presents with optic disc edema [39]. Most likely, it is caused by a state of hypercoagulability and leukocytosis, although direct leukemic infiltration could also be involved in its pathogenesis [37].

Optic nerve infiltration can be observed in acute leukemias and, more rarely in chronic forms [52,53]. The optic disc presents as pale and swollen with blurred margins, and may be accompanied by hemorrhages [52]. Optic nerve infiltration is frequent in children affected by acute leukemias and should rain concern for CNS involvement [54,55,56]. 

In patients with lymphocytic leukemia, iris infiltration is rare but may be the first sign of relapse [57,58]. Patients may present with blurred vision, conjunctival injection, anterior chamber reaction, pseudohypopyon, thickening of the iris stroma, change in iris shape and color are common clinical signs in leukemic iris infiltration [59]. 

More unusual ocular manifestations of leukemia include chronic conjunctivitis with redness, discharge and follicle-like lesions of the upper and lower palpebral conjunctiva in lymphocytic leukemia [60], corneal ring ulcer in acute monocytic leukemia [61], Sjogren’s syndrome in chronic lymphocytic leukemia [62] anterior segment ischemia in chronic myelogenous leukemia [63].

Secondary intraocular lymphomas affect the eye from a lymphoma originating outside the CNS. Most cases involve orbital lymphomas that extend directly into intraocular structures or non-Hodgkin lymphomas (NHL) that spread to the eye through the bloodstream, affecting the uvea, ocular adnexa, orbit, lacrimal gland, and conjunctiva [64]. Intraocular NHL can produce diffuse or multiple vitreoretinal foci as well as unifocal or multifocal lesions to the uvea, with variable presentations [65]. Iris lymphoma, for instance, tends to be high-grade and usually develops in patients with known aggressive systemic disease [66].

## 4. Ocular GVHD

Ocular GVHD (oGVHD) is a form of chronic GVHD predominantly affecting the ocular surface. It impacts approximately 40–60% of individuals who undergo allogeneic HSCT [67]. The incidence of oGVHD is higher in cases of chronic GVHD affecting other organs, with 50–90% of patients experiencing systemic GVHD also presenting with symptoms of oGVHD [68,69]. 

The pathophysiology of oGVHD is rapidly progressive, with inflammatory and donor T cell-mediated immune dysregulatory mechanisms leading to fibrosis of the lacrimal gland, conjunctiva and meibomian glands, possibly with corneal involvement [70]. 

The lacrimal gland is the principal target of oGVHD. Donor CD4+ T cells and CD8+ T cells infiltrate the preductal area of the gland, generating a pro-inflammatory environment and thus recruiting macrophages, antigen-presenting cells (APCs), and CD34+ stromal fibroblasts [71,72]. Recent evidence suggests that recruited fibroblasts may either derive from circulating donor-derived precursors or may arise from local epithelial to mesenchymal transition [73,74]. Regardless of their source, the accumulation of abnormal collagen fibers and extracellular matrix components by fibroblasts results in the fibrosis of the glandular interstitium [5,75]. These alterations ultimately result in lacrimal gland dysfunction, with consequent aqueous deficient dry eye. 

**Figure 1:**
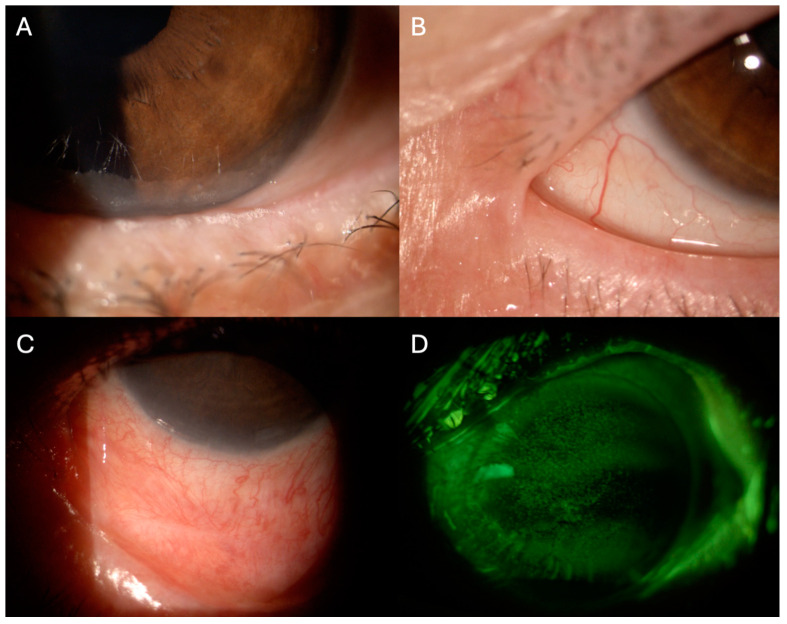
Slit-lamp photographs of clinical findings of ocular graft versus host disease. (**A**). Foamy secretion from meibomian gland dysfunction. (**B**) Ankyloblepharon, (**C**). Symblepharon. (**D**) Corneal punctate epithelial defects were highlighted using fluorescein staining with cobalt blue and yellow filters.

Meibomian gland dysfunction (MGD) is observed in 47.8% of oGVHD, indicating that affected patients may present both evaporative and aqueous-deficient forms of dry eye [76]. The pathophysiological mechanisms leading to MGD after HSCT remain elusive, and recent data highlights that some degree of glandular dysfunction may already exist prior to the transplantation process [77,78]. The main pathologic features observed in affected patients include obstruction of the gland orifice and cystic dilations of the ducts with atrophy. This process is mediated by lymphocyte infiltration and fibrosis around the glandular structures, resulting in altered meibum secretion and consequent tear film instability (Figure 1A) [79,80]. 

Conjunctival tissue involvement is observed in approximately 10% of oGVHD and is considered a negative prognostic factor for overall survival, being an indicator of severe systemic involvement [69,81]. Histopathological studies have evidenced the presence of lymphocytic infiltrating of the subconjunctival stroma, as well as neutrophils and neutrophil extracellular traps [82,83,84]. The complex interaction of these players contributes to the changes observed in the conjunctival tissue of oGVHD, such as fibrosis with goblet cell metaplasia, shortening, and reduced density of microvilli [85]. Clinically, these findings translate into conjunctival hyperemia, chemosis, and cicatricial conjunctivitis with consequent lagophthalmos, punctal stenosis, subtarsal fibrosis, symblepharon, and ankyloblepharon (Figure 1B,C) [86,87]. Superior limbal keratoconjunctivitis-like inflammation may also be present [88].

Such ocular surface alterations may induce corneal suffering. Corneal in vivo confocal microscopy findings have revealed increased density of dentritic epithelial cells and globular immune cells with altered morphology of corneal sub-basal nerves [89,90]. Clinically, superficial punctate keratopathy is the most frequent finding (Figure 1D) but neovascularization and sterile ulceration have been reported in severe cases [86,91]. Patients report pain, photophobia and decreased vision.

To date, diagnosis of oGVHD relies on two international criteria: National Institutes of Health (NIH) guidelines and the International Consensus Group of ophthalmologists’ criteria (Table 1) [92,93]. 

Treatment of oGVHD has different objectives: (i) lubrication, (ii) control of drainage, (iii) control of evaporation, and (iv) decrease of ocular surface inflammation. The main therapeutic tools include permanent or reversible punctual occlusion, eyelid hygiene with warming compresses, and lid margin cleansing [94]. In severe cases, topical antibiotic ointments, corticosteroids, or cyclosporin may help obtain disease remission, as well as systemic fatty acid supplementation and systemic tetracyclines [4]. 

## 5. Toxicity to Antineoplastic Therapies

**Figure 2:**
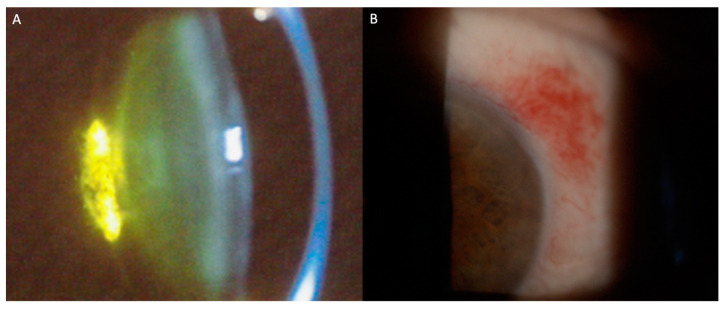
Slit-lamp photographs of signs of ocular toxicities to antineoplastic agents. (**A**) Posterior subcapsular cataract; (**B**) Conjunctival hemorrhage.

Systemic chemotherapy and targeted therapies used in leukemia treatment may cause significant ocular morbidity. Frequent findings include posterior subcapsular cataracts and subconjunctival hemorrhages (Figure 2A,B). Table 2 represents the most frequent ocular findings in patients treated with systemic agents. 

Eye radiation therapy may cause self-limiting acute toxicity as periorbital erythema, eyelash loss, conjunctival injection, excessive tearing, and swelling, with a higher incidence in dosages >25 Gy [95,96]. Chronic toxicity is observed in 50% of treated patients and includes the development of cataracts, dry eye disease, retinopathy, and keratitis [97]. 

PVRL may sometimes be treated using intravitreal injections of chemotherapies or immunotherapies. The most commonly reported complications include elevations in intraocular pressure and epithelial keratopathy [17].

**Table 2:** Ocular toxicity from systemic anti-neoplastic therapy.

Agent	Sign/Symptom	Underlying Mechanism
Busulfan [98,99,100]	Posterior subcapsular cataracts	Inhibition of nucleic acid formation in lens epithelium
Vincristine [101]	Temporary or permanent loss of vision	Axonal damage due to microtubule disruption
Dexamethasone [102]	Increased intraocular pressure; cataract	Increased resistance to aqueous outflow; unclear, likely non-enzymatic formation of Schiff base intermediates
Fludarabine [103,104]	Rapid progressive loss of vision	Direct damage to retinal bipolar and ganglion cells; gray and white matter
Cytarabine [105,106]	Reversible corneal toxicity and conjunctivitis	Unknown, likely inhibition of DNA synthesis corneal and conjunctival epithelium
Imatinib [107]	Periorbital edema, conjunctival hemorrhage	Periocular soft tissue expression of molecular targets for Imatinib
Immune checkpoint inhibitors [108]	Uveitis	Activation of complement cascade, recruitment of innate immunity cells in cerebrospinal fluid, loss of immune-privilege

## 6. Conclusions

Ocular complications are a critical yet often underrecognized aspect of managing hematological malignancies. The eye can be a barometer for systemic disease activity and adverse effects of treatment. As such, routine ophthalmic assessments should be integrated into the standard of care for these patients. Interdisciplinary collaboration between hematology and ophthalmology services is vital for prompt identification and management of ocular issues. Advances in targeted cancer therapies hold promise for reducing ocular side effects and enhancing patient quality of life. Awareness of ocular symptoms, both within medical teams and amongst patients, is key to safeguarding vision and ensuring comprehensive care in the context of blood cancers. 

## Figures and Tables

**Table 1:** Elements for ocular GVHD diagnosis according to the NIH diagnostic criteria and to the International Consensus Group of ophthalmologists’ criteria.

National Institutes of Health (NIH) Diagnostic Criteria	International Consensus Group of Ophthalmologists’ Criteria
Schirmer’s test mean value of ≤5 mm at 5 min	Pathological ocular surface disease index (OSDI) questionnaire (<13)
New onset of keratoconjunctivitis sicca as determined by a slit lamp examination, with a mean Schirmer’s test value of 6 to 10 mm	Pathological Schirmer’s test (<10 mm in 5 min) without anesthesia
	Positive corneal fluorescein staining
	Presence of conjunctival injection

## Data Availability

No new data were created in this study.
